# Anti-Inflammatory Effects of *Cordyceps* Cs-HK1 Fungus Exopolysaccharide on Lipopolysaccharide-Stimulated Macrophages via the TLR4/MyD88/NF-κB Pathway

**DOI:** 10.3390/nu16223885

**Published:** 2024-11-14

**Authors:** Yan-Yu Zhu, Yu-Han Dong, Fang-Ting Gu, Zi-Chen Zhao, Lin-Xi Huang, Wai-Yin Cheng, Jian-Yong Wu

**Affiliations:** Research Institute for Future Food, Department of Food Science and Nutrition, The Hong Kong Polytechnic University, Hung Hom, Kowloon 999077, Hong Kong

**Keywords:** polysaccharide, anti-inflammatory activity, macrophages, TLR4/MyD88 signaling pathway, reactive oxygen species

## Abstract

Chronic inflammation is a common factor in the pathological processes of multiple human diseases. EPS-LM, an exopolysaccharide (EPS) from the Cordyceps sinensis fungus Cs-HK1, has shown notable anti-inflammatory activities in previous studies. This study aimed to investigate the major signaling events mediating the anti-inflammatory effects of EPS-LM in lipopolysaccharide (LPS)-stimulated RAW 264.7 macrophage cell culture. EPS-LM treatment significantly reduced LPS-induced production of pro-inflammatory mediators, including nitric oxide (NO) and reactive oxygen species (ROS). It also suppressed the expression levels of Toll-like receptor 4 (TLR4) and myeloid differentiation primary response gene 88 (MyD88), subsequently delaying the translocation of nuclear factor-kappa B (NF-κB) to the nucleus. Additionally, co-immunoprecipitation (Co-IP) experiments demonstrated that EPS-LM inhibited the binding of TLR4 to MyD88. The ability of EPS-LM to inhibit the TLR4/MyD88/NF-κB pathway, coupled with its capacity to reduce oxidative stress, underscores its multifaceted anti-inflammatory effects. These effects render EPS-LM as a promising candidate for the comprehensive management of various inflammatory and oxidative stress-related conditions, protecting against cell damage.

## 1. Introduction

Inflammation is a fundamental biological response in the human body to protect against harmful stimuli, such as infections, injuries, and toxins. It plays a critical role in initiating defense mechanisms and facilitating tissue repair. Inflammation is beneficial and essential for recovery. However, when the inflammatory response becomes dysregulated, it can lead to pathological conditions. Chronic inflammation is implicated in the progression of numerous diseases, including autoimmune disorders (e.g., rheumatoid arthritis, lupus), cardiovascular diseases (e.g., atherosclerosis), neurodegenerative disorders (e.g., Alzheimer’s disease), and various other chronic inflammatory conditions [1,2]. Given the public health impact associated with these diseases, there is an urgent need for novel therapeutic strategies for effectively modulating the inflammatory responses and preventing or treating inflammation-related pathologies.

In the search for effective therapeutic options, natural products with anti-inflammatory properties have garnered considerable attention. Among these, *Cordyceps sinensis* (or *Ophiocordyceps sinensis*), generally known as the Chinese caterpillar fungus, has been widely recognized in traditional Chinese medicine (TCM) for its therapeutic benefits. Traditionally, *C. sinensis* was used to treat a variety of diseases, including respiratory disorders, chronic fatigue, and kidney dysfunction, with its primary functions in TCM being to “tonify the kidneys and lungs” and alleviate symptoms of chronic inflammation [3]. Recent studies from the authors’ group have been focused on the exopolysaccharides (EPS) produced by the mycelial fermentation of a *C. sinensis* fungus Cs-HK1 [4]. The Cs-HK1 fermentation process has been optimized to produce high yields of bioactive EPS, which are secreted by the fungus as part of its extracellular matrix. The EPS was further separated into two major fractions, EPS-HM and EPS-LM, based on differences in molecular weight and chemical composition [5]. Among these, EPS-LM, the lower molecular weight fraction, has demonstrated significantly higher anti-inflammatory activity. Specifically, EPS-LM has been shown to inhibit lipopolysaccharide (LPS)-induced pro-inflammatory responses in macrophage cell cultures. This is particularly important as LPS is commonly used to induce inflammation in vitro. EPS-LM has been shown to effectively suppress the activation of nuclear factor kappa B (NF-κB), a transcription factor that regulates the production of key pro-inflammatory mediators, including nitric oxide (NO), tumor necrosis factor-alpha (TNF-α), and interleukin-8 (IL-8) [6]. Given the high bioavailability of EPS-LM and potent inhibitory effects on inflammation, it was selected for further investigation in the present study. This study aims to explore the molecular mechanisms by which EPS-LM exerts its anti-inflammatory effects.

In addition to *C. sinensis*, polysaccharides from other medicinal fungi and plants have demonstrated significant anti-inflammatory effects. For instance, polysaccharides from *Ganoderma lucidum* (GLP) and *Angelica sinensis* (ASP-Ag-AP) have been shown to modulate the TLR4/MyD88/NF-κB signaling pathway, a key mediator of inflammatory responses. GLP has been demonstrated to protect against colitis and tumorigenesis by reducing the production of pro-inflammatory cytokines such as IL-1β, inducible nitric oxide synthase (iNOS), and cyclooxygenase-2 (COX-2) [7]. Furthermore, GLP enhances gut barrier function and improves microbial dysbiosis, further contributing to its anti-inflammatory effects. Similarly, ASP-Ag-AP has demonstrated strong protective benefits in animal models of colitis, reducing inflammation and modulating gut microbiota and barrier function [8]. These studies highlight the therapeutic potential of natural polysaccharides in managing inflammation by targeting key molecular pathways.

Toll-like receptor 4 (TLR4) acts as a transmembrane pattern-recognition receptor capable of responding to various pathogen-associated and damage-associated molecular patterns [9,10]. Upon activation, TLR4 engages myeloid differentiation factor 88 (MyD88), initiating a signaling cascade that leads to the activation of NF-κB. This activation results in the upregulated transcription of genes encoding inflammatory cytokines. Furthermore, the TLR4/MyD88/NF-κB signaling pathway is essential in facilitating the inflammatory responses induced by LPS, a constituent of the outer membrane of Gram-negative bacteria. Activation of this pathway results in the synthesis of pro-inflammatory mediators, including NO and reactive oxygen species (ROS). These mediators play a crucial role in the signaling pathways of the human immune response but may also cause adverse inflammatory effects and tissue damage if generated excessively [11,12]. Therefore, the TLR4/MyD88/NF-κB signaling pathway is a common therapeutic target for anti-inflammatory natural products, including natural polysaccharides [8,13].

This study aimed to investigate the anti-inflammatory effects of EPS-LM on LPS-stimulated macrophages through the TLR4/MyD88/NF-κB signaling pathway. Based on the background above, we hypothesize that EPS-LM modulates this signaling pathway, thereby exerting anti-inflammatory effects as shown in our previous studies [6]. Experiments were performed in RAW 264.7 macrophage cell culture, which is one of the most popular in vitro models for immunomodulatory and anti-inflammatory studies.

## 2. Materials and Methods

### 2.1. Cs-HK1 Mycelial Fermentation and EPS-LM Isolation

**Figure 1:**
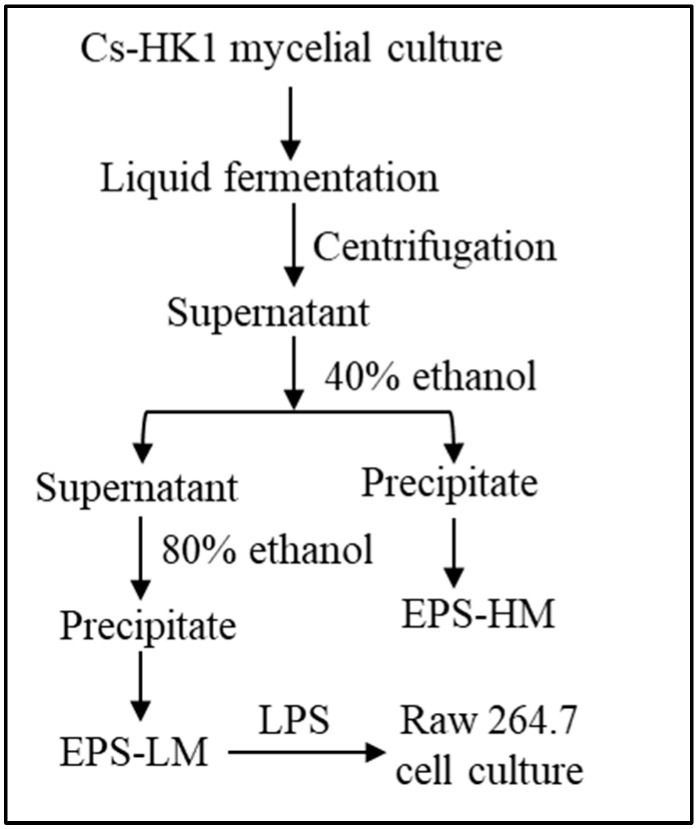
Major experimental steps for isolation of EPS-LM from Cs-HK1 mycelial fermentation and purification process.

The mycelial fermentation of Cs-HK1 was conducted in shake flasks to produce EPS-LM. As reported previously [5,14], the liquid medium contained 40 g/L glucose, 5 g/L peptone, 1 g/L KH_2_PO_4_, 0.5 g/L MgSO_4_·7H_2_O, and 10 g/L yeast extract. The culture flasks were incubated at 20 °C and 200 rpm for 7 days and the fermentation liquid was then centrifuged at 12,000 rpm for 20 min at 4 °C. The liquid supernatant was collected and subjected to two-step ethanol precipitation [6], using 40% (*v*/*v*) of ethanol in the first step and 80% (*v*/*v*) ethanol in the second step, as illustrated in Figure 1. The precipitates were recovered from the liquid mixture by centrifugation at 10,000 rpm for 20 min. The precipitates were re-dissolved in water and lyophilized following centrifugation and collected as the crude EPS fractions, EPS-HM in the higher MW range from the first step and EPS-LM in the lower MW range from the second step.

### 2.2. Analysis of EPS-LM Composition and Properties

#### 2.2.1. Analysis of the Chemical Composition

The total carbohydrate and protein content of EPS-LM were determined by the Anthrone test and by the Lowry method, respectively. As reported previously [15,16], the EPS samples were hydrolyzed at 100 °C with anthrone reagent in 80% H_2_SO_4_ and the absorbance of the sample solutions was measured at 620 nm, and converted to total carbohydrate by calibration with glucose as a reference standard. The protein content was quantified by measuring absorbance at 750 nm, using bovine serum albumin (BSA) as the reference standard.

The monosaccharide composition of EPS-LM was analyzed by PMP-HPLC, following the previously published procedure [6]. In brief, the EPS-LM sample was hydrolyzed in 2 M TFA at 110 °C for 4 h, followed by vacuum evaporation to complete dryness. The remaining solid was redissolved in de-ionized water and mixed with equal volumes of 0.5 M PMP solution in methanol and 0.3 M NaOH solution, then incubated at 70 °C for 30 min. The reaction was stopped by adding 0.3 M hydrochloric acid, then rinsed three times with chloroform, and the aqueous layer was collected for high-performance liquid chromatography (HPLC) analysis. An Agilent Zorbax Eclipse XDB-C18 column (150 mm × 4.6 mm) was used for the analysis on an Agilent 1100 instrument (Agilent Technologies, Santa Clara, CA, USA) at a temperature of 25 °C. The mobile phase comprised a potassium phosphate-buffered saline solution (0.05 M, pH 6.9) with 15% acetonitrile (solution A) and 40% acetonitrile (solution B). Each sample was injected at 20 μL to the system detected with UV at 250 nm. The specific monosaccharide peaks were identified and quantified by calibration with monosaccharide standards (Sigma, St. Louis, MO, USA).

#### 2.2.2. Analysis of the Molecular Weight

The MW of EPS-LM was measured using high-performance size-exclusion chromatography (HPSEC) with detection by refractive index (RI) and multi-angle laser light scattering (MALLS), as reported previously [16]. The instrumental system consisted of a Waters 1515 isocratic pump, two HPSEC columns (SB-806 HQ and SB-804 HQ, 7.8 × 300 mm, Shodex, Tokyo, Japan), a 2414 RI detector (Waters Co., Milford, MA, USA), and a MALLS detector (DAWN HELEOS II, Wyatt Technology, Goleta, CA, USA). An aliquot of 100 μL sample solution was injected into the system and eluted with water as the mobile phase at a flow rate of 0.5 mL/min. The average MW of the EPS-LM fractions was calculated using a refractive index increment (dn/dc) value of 0.138 mL/g. Data collection and analysis were conducted using Astra software (version 6.1.7, Wyatt Technologies Co., Santa Barbara, CA, USA).

#### 2.2.3. FT-IR Analysis

An infrared (IR) spectroscopic analysis was performed at room temperature using an Avatar 360 Fourier-transform infrared (FT-IR) spectrometer (Thermo Nicolet, Cambridge, UK). The analysis covered the spectral range of 4000–500 cm^−1^ with a resolution of 4 cm^−1^. The solid EPS-LM was pulverized with potassium bromide (KBr) powder and pressed into a pellet for FT-IR analysis [15].

### 2.3. Assessment of EPS-LM Bioactivities

#### 2.3.1. RAW 264.7 Macrophage Cell Culture

The in vitro anti-inflammatory activity assays were performed using LPS-stimulated RAW 264.7 macrophage cell cultures [5]. Dulbecco’s Modified Eagle Medium (DMEM), supplemented with 10% fetal bovine serum (FBS), 100 U/mL penicillin, and 100 µg/mL streptomycin, all obtained from Gibco Biotechnology Co., Ltd. (Grand Island, NY, USA), was used as the base culture medium. All other agents related to cell culture, including trypsin, were also obtained from Gibco. The cell cultures were maintained at 37 °C in a humidified incubator with 5% CO_2_.

#### 2.3.2. Reagents and Antibodies

Antibodies used included MyD88 (4283S, CST), NF-κB p65 (#8242, CST), interleukin-18 (IL-18) (A1115, ABclonal, Woburn, MA, USA), TLR4 (Sc-293072, Santa Cruz, Santa Cruz, CA, USA), and β-actin (#4970, CST, Danvers, MA, USA). Additionally, horseradish peroxidase (HRP)-conjugated goat anti-rabbit (A0208, Beyotime, Shanghai, China) and HRP-conjugated goat anti-mouse (A0216, Beyotime) were utilized. LPS from Escherichia coli 0111: B4, along with reagents such as 3-(4,5-Dimethylthiazol-2-yl)-5-(3-carboxymethoxyphenyl)-2-(4-sulfophenyl)-2H-tetrazolium (MTS), 2,7-Dichlorodihydrofluorescein diacetate (DCFH-DA, Shanghai, China), sulphanilamide, N-1-naphthyl ethylenediamine dihydrochloride, and phosphoric acid, were sourced from Sigma-Aldrich Co., Ltd. (St. Louis, MO, USA). Phenazine methosulfate (PMS) was obtained from Promega Corporation (Madison, WI, USA).

#### 2.3.3. Cell Viability Assay

Cell viability was assessed using the MTS tetrazolium compound and the electron coupling reagent PMS, following the documented procedure [17]. In metabolically active cells, dehydrogenases convert MTS into a water-soluble formazan, which can be quantified by measuring absorbance. RAW 264.7 cells in the logarithmic growth phase were seeded into 96-well plates (100 μL/well) at a concentration of 2 × 10^5^ cells/mL and incubated for 24 h. The cells were then treated with EPS-LM at concentrations of 25, 50, 100, and 200 μg/mL and incubated for another 24 h. The absorbance of the resulting formazan was measured at 490 nm, with all operations conducted without exposure to light.

#### 2.3.4. NO Assay

The amount of NO formed was estimated from the accumulation of the stable NO metabolite, nitrite (NO_2_^−^), using the Griess assay. Cells were inoculated in 96-well plates at a density of 2 × 10^5^ cells/mL, with culture conditions and treatments as described in Section 2.1. A reagent mixture of 1% (*w*/*v*) sulfanilamide, 0.1% (*w*/*v*) naphthyl ethylenediamine, and 2% (*v*/*v*) phosphoric acid was prepared. Samples were incubated with these reagents separately for 10 min at 25 °C, and optical density (OD) values were measured at 540 nm [18]. The amount of nitrite was calculated from a NaNO_2_ standard curve (0–100 µM).

#### 2.3.5. Intracellular ROS Assay (DCFH-DA Probe)

Intracellular ROS levels were quantified using the method described by [19]. DCFH-DA is a cell-permeant fluorogenic dye that measures hydroxyl, peroxyl, and other ROS activities in cells. Cells were seeded in 96-well plates at a density of 2 × 10^5^ cells/mL, following the culture conditions and treatments outlined in Section 2.1. DCFH-DA is deacetylated by cellular esterases into a non-fluorescent compound, which is subsequently oxidized by ROS into 2’,7’-dichlorofluorescein (DCF). DCF fluorescence is detected with excitation at 485 nm and emission at 527 nm using a microplate reader (Molecular Devices SpectraMax Gemini X, Ramsey, MN, USA).

#### 2.3.6. Western Blotting Analysis

The Western blot approach was used to identify the expression of inflammation-related proteins, as reported by Li et al., with minor adjustments [6]. After treatment with LPS and EPS-LM, RAW 264.7 cells were collected and washed with cold phosphate-buffered saline (PBS, pH 7.4). Ice-cold cell lysis reagent (Catalogue #R0278, Sigma-Aldrich, Steinheim, Germany) was used to extract total cytoplasmic proteins, including those for IL-18, NF-κB p65, TLR4, and MyD88 detection. Actin served as an internal reference. The protein content in the supernatant was measured using the bicinchoninic acid (BCA) protein assay kit (Catalogue #23225, Thermo Fisher Scientific, Rockford, IL, USA). The lysate protein (20 µg per lane) was separated using 10% SDS-PAGE and transferred to a polyvinylidene fluoride (PVDF) membrane. The membrane was blocked with 5% skimmed milk (*w*/*v*) and treated with specific primary antibodies overnight at 4 °C, followed by the addition of secondary antibodies coupled with horseradish peroxidase. The blots were then developed using enhanced chemiluminescence and visualized via autoradiography.

#### 2.3.7. Immunofluorescence and Confocal Microscopy Measurement

After treatment with LPS and EPS-LM for 24 h, RAW 264.7 cells were fixed with 4% paraformaldehyde at room temperature for 10 min. The cells were then treated with 0.1% Triton X-100 for 5–10 min at room temperature, followed by three washes with PBS. To block nonspecific antibody binding, the cells were incubated with 1% BSA in PBS for 30 min. After blocking, the cells were incubated with diluted primary antibodies against MyD88, TLR4, and NF-κB p65 at 4 °C for 1 h. The cells were washed 3–5 times with PBS for 5 min each to remove unbound primary antibodies. Next, they were incubated with diluted fluorescein isothiocyanate (FITC)-conjugated secondary antibodies at room temperature for 1 h in the dark. The cells were washed three times with PBS for 5 min each to remove unbound secondary antibodies. For nuclear visualization, the cells were treated with DAPI (4’,6-diamidino-2-phenylindole) for 5 min at room temperature and then washed 2–3 times with PBS. Images were visualized using confocal microscopy (Nikon Eclipse Ti2-E live-cell imaging system, Nikon Corporation, Tokyo, Japan).

#### 2.3.8. Co-Immunoprecipitation (Co-IP) Assay

Approximately 1.0 × 10^7^ macrophage cells were seeded in each 6 cm dish and exposed to LPS and/or EPS-LM for 24 h, followed by protein extraction as described above. Immunoprecipitation was performed with slight modifications to the previously reported method [20]. Protein A+G magnetic beads were prepared and incubated with antibodies against TLR4 and MyD88 or control immunoglobulin G (IgG), followed by washing and resuspension in Tris-buffered saline (TBS). The protein samples were incubated with antibody-bound beads overnight at 4 °C, washed, and eluted with SDS-PAGE sample buffer. The eluted proteins were separated by SDS-PAGE and transferred to a 0.45 μm PVDF membrane. Membranes were blocked, incubated with primary antibodies overnight, washed, and then incubated with secondary antibodies. Detection was performed using chemiluminescence, and images were captured and analyzed to assess the expression levels of TLR4 and MyD88.

### 2.4. Statistical Data Analysis

The data were analyzed using GraphPad Prism software version 10 (GraphPad Software Inc., La Jolla, CA, USA) with one-way analysis of variance (ANOVA) for multiple comparisons, followed by Tukey’s post hoc test. All numerical data are expressed as the mean ± standard deviation (SD) of at least three independent experiments. The significance level was set at *p* < 0.05.

## 3. Results

### 3.1. EPS-LM Composition and Effect on RAW 264.7 Cell Viability

As shown in Table 1, the analysis of EPS-LM presented a molecular weight of 6.58 × 10^5^ Da, a sugar content of 18.7 wt%, and a high protein level of 30.0 wt%. The MW was consistent with our previous study on EPS-LM [6], which showed a significant peak at 3.60 × 10^5^ Da. Moreover, the EPS-LM in the previous study had a higher total carbohydrate content of 25.5 wt% and a lower protein content of 20.1 wt%. Both studies indicated that glucose and mannose were the main monosaccharide constituents of EPS-LM, but in the present study, mannose was more abundant. Additionally, the present study showed the existence of rhamnose and glucosamine at very low levels, which were not mentioned in the previous study. In general, EPS-LM exhibited a consistently high molecular weight and major monosaccharide composition, proving the stability of the fermentation process.

**Figure 2:**
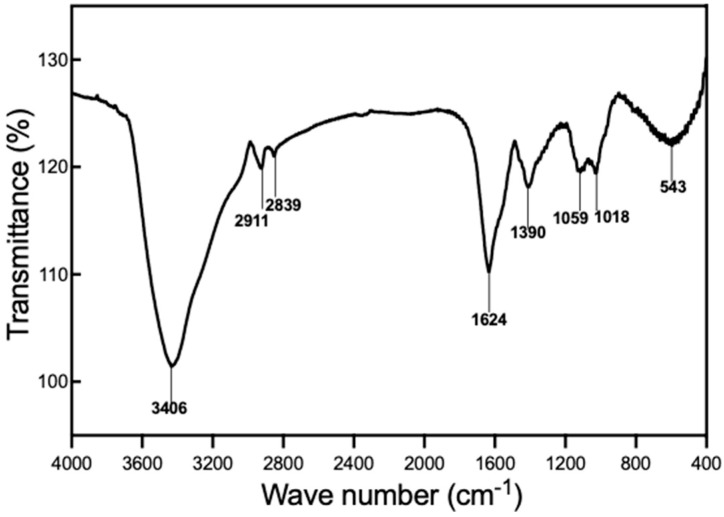
FT-IR spectrum of EPS-LM isolated from Cs-HK1 mycelial fermentation medium.

Figure 2 shows the FT-IR spectrum of EPS-LM, which reveals several characteristic peaks. The broad and intense peak around 3400 cm^−1^ is attributed to the axial stretch of the -OH group in the glycol chain, indicating the presence of numerous hydroxyl groups commonly associated with polysaccharides. Additionally, the peak around 1390 cm^−1^ is due to OH bending vibration, further confirming the abundance of hydroxyl groups in EPS-LM. A weak peak around 2911 cm^−1^ corresponds to C-H stretching vibration, suggesting the presence of organic compounds with C-H groups. The peak at 1624 cm^−1^ is characteristic of asymmetric and symmetric vibrations of the ring stretching of the carboxylate group, indicating the presence of carboxylate groups potentially related to the anti-inflammatory and antioxidant properties of EPS-LM. Absorption peaks in the region of 1000–1200 cm^−1^ are associated with the stretching vibrations of C-O-H side groups and C-O-C glycosidic bond vibrations, which are indicative of glycosidic linkages and side groups typical of polysaccharide structures. The peak around 1059 cm^−1^ is attributed to C-O stretching of pyranoside, particularly in glucose residues. The absence of an absorption peak around 1710–1740 cm^−1^ confirms the absence of carboxylic acid groups in EPS-LM. The characteristics of EPS-LM observed in this study are similar to those reported in previous research on EPS-LM produced by Cs-HK1. The FT-IR spectrum shows analogous peaks, suggesting that both studies describe EPS-LM with certain structural features that could potentially contribute to their comparable biological activities [6].

**Figure 3:**
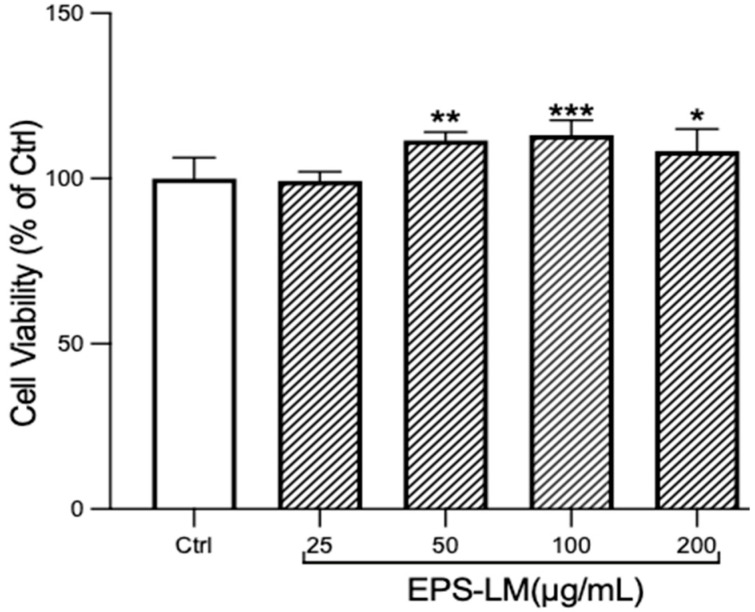
Effects of EPS-LM treatment on cell viability and proliferation in RAW 264.7 cells. (Data as mean ± SEM, *n* = 6. One-way ANOVA followed by Tukey’s multiple comparison test: * *p* < 0.1, ** *p* < 0.01, *** *p* < 0.001 versus Ctrl).

As shown in Figure 3, the OD values of the EPS-LM treated groups show no significant difference from the control in the concentration range of 25–200 μg/mL. This indicates that EPS-LM is non-toxic to RAW 264.7 cells and promotes cell growth, which could be regarded as an immunostimulatory effect of the EPS-LM.

### 3.2. EPS-LM Reduces ROS and Inhibits Oxidative Stress Induced by LPS

**Figure 4:**
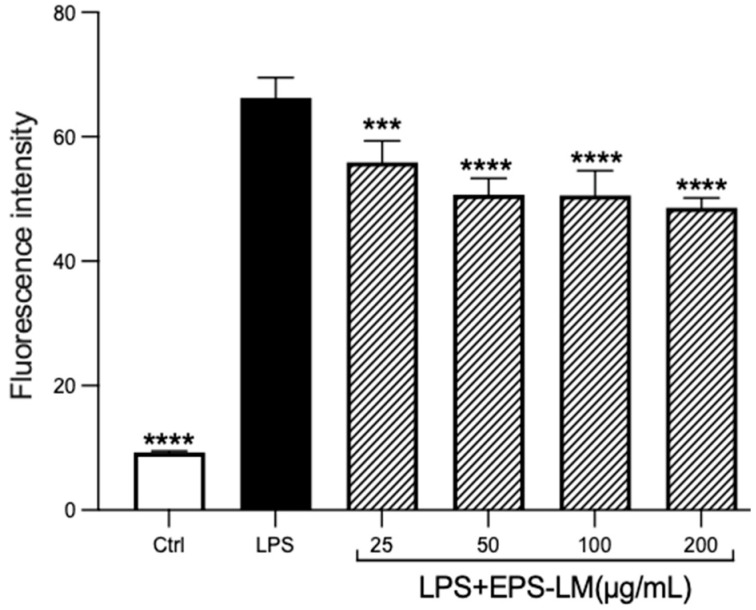
Effects of EPS-LM on LPS-induced ROS production in RAW 264.7 cells. (Data as mean ± SEM, *n* = 6. One-way ANOVA followed by Tukey’s multiple comparison test: *** *p* < 0.001, **** *p* < 0.0001 versus LPS).

As shown in Figure 4, LPS treatment caused a dramatic increase in the ROS level (proportional to the fluorescence intensity) of macrophage cells compared with the control group. The addition of EPS-LM together with LPS, denoted as LPS+EPS-LM, significantly lowered the LPS-stimulated ROS production. The increase in EPS-LM concentration from 25 to 200 μg/mL resulted in a more substantial suppression of ROS production. These results suggest that EPS-LM can effectively reduce the LPS-stimulated ROS production in RAW 264.7 cells.

### 3.3. EPS-LM Effects on LPS-Induced Morphological Changes and NO Production

**Figure 5:**
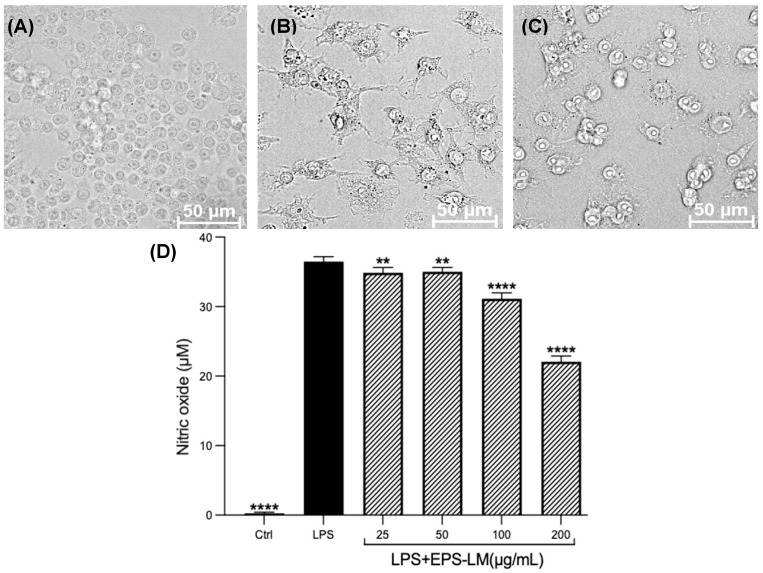
Effects of LPS and EPS-LM on RAW 264.7 cells: morphology of (**A**) control; (**B**) LPS-treated; (**C**) LPS + 200 µg/mL EPS-treated group; (**D**) NO production. (Data as mean ± SEM, *n* = 6; One-way ANOVA followed by Tukey’s multiple comparison test: ** *p* < 0.01, **** *p* < 0.0001 versus LPS).

LPS treatment of the macrophage cells also altered their morphology (Figure 5A–C). While cells in the control group exhibited a uniform and oval shape, the LPS-treated cells showed obvious differentiation into a shuttle shape. This morphological change, combined with the results of the NO assay, indicates successful induction of an inflammatory response and establishment of the inflammation model. As shown in Figure 5D, EPS-LM significantly suppressed the LPS-induced production of NO. At the maximum concentration tested (200 µg/mL), EPS-LM decreased the LPS-stimulated production of NO by about 40%. The inhibitory effects of EPS-LM on NO production followed a dose-dependent trend in the range of 25–200 µg/mL.

### 3.4. EPS-LM Inhibits the TLR4/MyD88/NF-κB Pathway to Mitigate Inflammation

**Figure 6:**
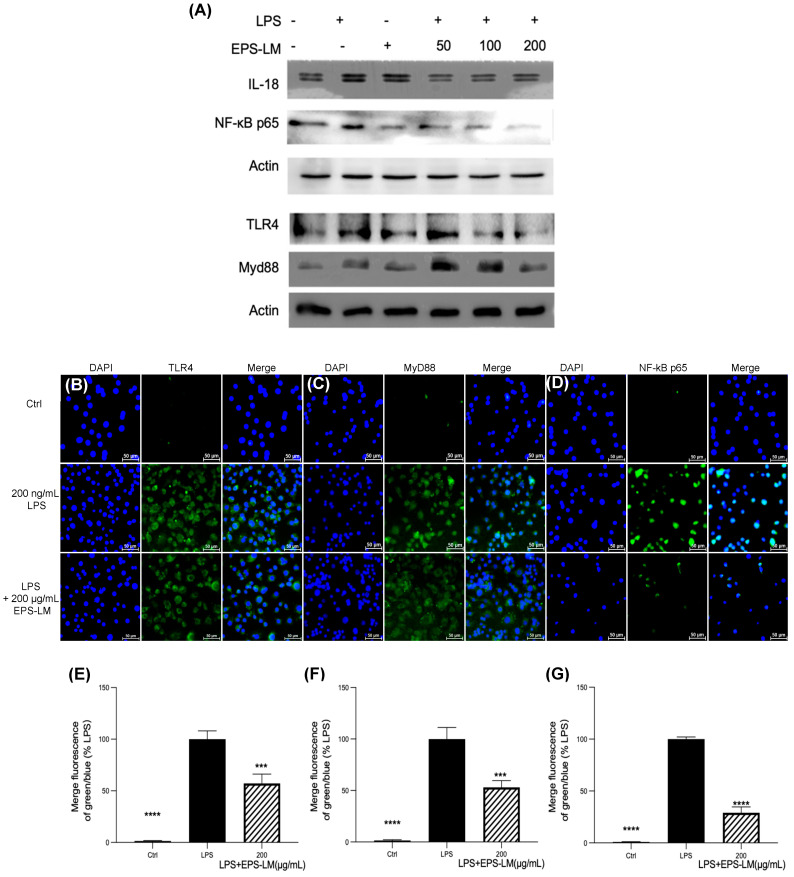
Western blotting and immunofluorescence analysis of the effect of EPS-LM on LPS-induced inflammatory protein expression in RAW 264.7 cells: (**A**) the representative bands of IL-18, NF-κB p65, TLR4, and MyD88 were determined by Western blotting. Actin were used as internal control; (**B**–**D**) TLR4, MyD88, NF-κB p65 expression levels in RAW 264.7 cells; (**E**–**G**) statistical analysis of the ratio of green to blue fluorescence for images corresponding to TLR4, MyD88, and NF-κB p65, respectively. (Data as mean ± SEM, *n* = 3. One-way ANOVA followed by Tukey’s multiple comparison test: *** *p* < 0.001, **** *p* < 0.0001 versus LPS).

Figure 6 shows all the results from Western blotting and immunofluorescence labeling assessment of the expression of critical proteins in the TLR4/MyD88/NF-κB signaling pathway. The Western blotting results (Figure 6A) show that LPS significantly increased the expression levels of IL-18, TLR4, NF-κB p65, and MyD88 proteins in RAW 264.7 cells, validating its effectiveness in activating the TLR4/MyD88/NF-κB pathway and eliciting an inflammatory response. Treatment of the cells with EPS-LM dramatically reduced the expression levels of these proteins in LPS-stimulated cells, indicating a substantial inhibitory effect on this pathway.

The immunofluorescence labeling assay provided further evidence for the changes in cellular localization and expression of these signaling molecules. The fluorescence intensity of TLR4 and MyD88 significantly increased after LPS exposure, indicating their heightened expression and involvement in the inflammatory process (Figure 6B,C). Additionally, LPS exposure prompted the nuclear translocation of NF-κB p65, a crucial step for initiating the transcription of inflammatory genes (Figure 6D). Treatment with EPS-LM significantly decreased the fluorescence intensity of both TLR4 and MyD88 after LPS exposure, demonstrating its potent capacity to suppress these critical inflammation-inducing proteins (Figure 6E,F). This suggests that EPS-LM inhibited both the expression and potential activation of these key inflammatory mediators. Furthermore, EPS-LM treatment notably reduced the nuclear translocation of NF-κB p65 (Figure 6G), indicating its role in obstructing the downstream signaling processes essential for initiating the transcription of pro-inflammatory genes. Therefore, these results provide support to the hypothesis that EPS-LM suppressed the LPS-induced inflammatory responses in the macrophage cells by blocking the TLR4/MyD88/NF-κB signaling pathway.

### 3.5. Effects of EPS-LM on the Interaction Between TLR4 and MyD88

**Figure 7:**
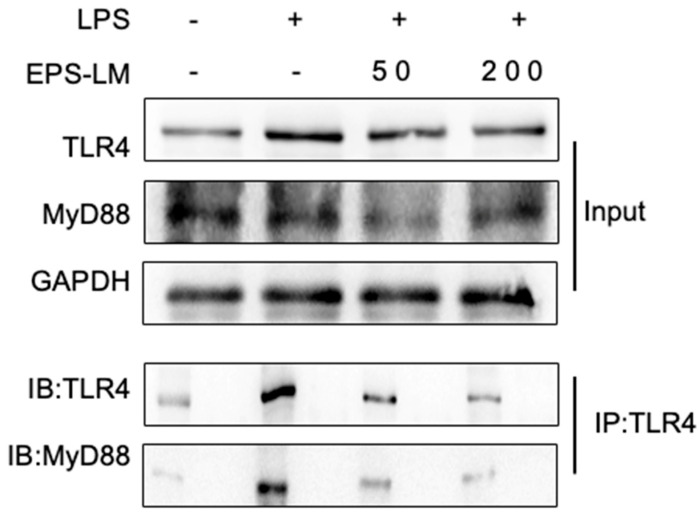
EPS-LM reduces the interaction between TLR4 and MyD88 in RAW 264.7 cells. RAW 264.7 cells were treated with LPS in the presence or absence of EPS-LM (50 and 200 µg/mL). Co-IP was performed to assess the interaction between TLR4 and MyD88. TLR4 and MyD88 were detected by immunoblotting (IB) in the input and TLR4-immunoprecipitated (IP: TLR4) samples. GAPDH was used as a loading control.

The treatment effects on the interaction between TLR4 and MyD88 were detected via co-immunoprecipitation (Co-IP). As shown in Figure 7, 24 h post-LPS stimulation, the contact between TLR4 and MyD88 increased considerably, suggesting that the TLR4/MyD88 signaling pathway had been successfully induced. However, EPS-LM significantly decreased the binding of TLR4 to MyD88, indicating that it efficiently disturbed their interaction. This result corroborated the above finding that EPS-LM suppressed the expression of TLR4 and MyD88 in LPS-stimulated RAW 264.7 cells, further proving the concept that EPS-LM exerts its anti-inflammatory effects by interfering with the TLR4/MyD88/NF-κB signaling pathway.

## 4. Discussion

The results demonstrate that EPS-LM significantly reduced NO and IL-18 levels in LPS-stimulated RAW 264.7 cells, indicating its potent in vitro anti-inflammatory activity. More specifically, EPS-LM inhibited the expression of TLR4, MyD88, and NF-κB proteins, which are critical components of the TLR4/MyD88/NF-κB signaling pathway, a key mediator of LPS-induced inflammatory responses. This downregulation reduces the generation of inflammatory mediators, suggesting a protective effect against LPS-induced cellular damage. By inhibiting the TLR4/MyD88/NF-κB signaling pathway, EPS-LM substantially reduces inflammation [8]. We further conducted experiments to investigate the interaction between TLR4 and MyD88. The Co-IP experiment was designed to elucidate the molecular interactions between TLR4 and MyD88 in the presence and absence of EPS-LM. After 24 h of LPS stimulation, Co-IP analysis revealed a significant increase in the interaction between TLR4 and MyD88, indicating successful activation of the TLR4/MyD88 signaling pathway. This interaction is a key step in the cascade that leads to the activation of NF-κB and the subsequent production of pro-inflammatory cytokines [21]. Interestingly, in cells co-treated with EPS-LM and LPS, the Co-IP results show a marked decrease in TLR4 binding to MyD88. This reduction suggests that EPS-LM effectively disrupts the critical connection between these two proteins, thereby impeding the signaling cascade at an early stage. By preventing the association of TLR4 with MyD88, EPS-LM inhibits the subsequent activation of NF-κB p65. By inhibiting its activation, EPS-LM significantly reduces the production of inflammatory cytokines, thereby mitigating the inflammatory response [22].

The findings from our present study are consistent with earlier research on comparable polysaccharides, confirming their anti-inflammatory capabilities. Both EPS-HM and EPS-LM, along with total EPS, have been demonstrated to decrease LPS-induced activation of NF-κB, a major transcription factor in the inflammatory response [6]. Attenuating NF-κB activation is critical because it induces the transcription of pro-inflammatory genes, including those encoding cytokines like TNF-α and interleukin-1 beta (IL-1β), which are pivotal in immunological and inflammatory responses, particularly during infection and trauma. The NF-κB signaling pathway stimulates cytokines, chemokines, and adhesion molecules, thereby controlling inflammation, immunity, and cell survival [23,24].

**Figure 8:**
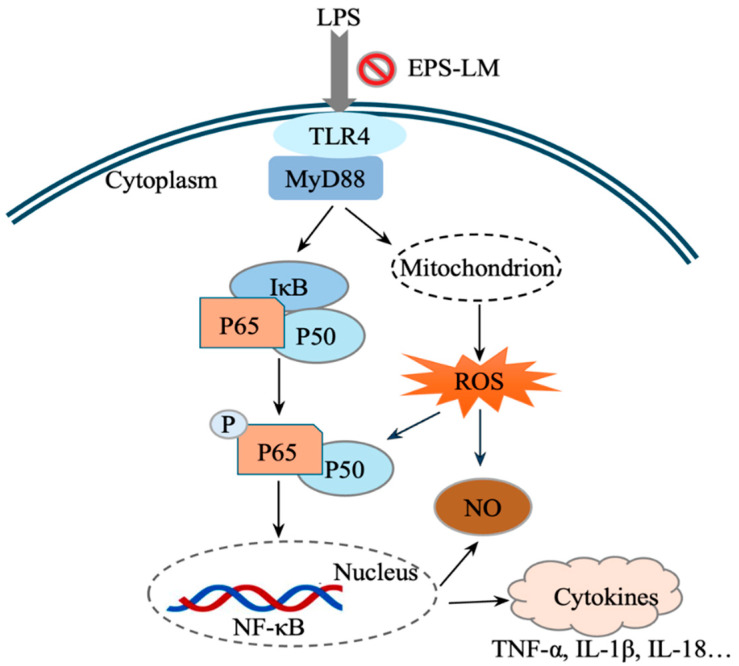
A hypothesized signal mechanism for the protective effects of EPS-LM against LPS-induced inflammatory injury and ROS via the TLR4/MyD88/NF-κB pathway: (1) LPS binding to TLR4 activates MyD88, leading to a signaling cascade that degrades IκB, releasing and phosphorylating p50 and p65 subunits; (2) these subunits form an active NF-κB complex that moves to the nucleus to transcribe pro-inflammatory cytokines like TNF-α and IL-1β; (3) LPS also induces ROS and NO production in mitochondria, contributing to inflammation and damage; (4) EPS-LM inhibits TLR4-MyD88 interaction, reducing ROS and NO generation and providing anti-inflammatory protection.

Our experiments, building on previous studies [6], further demonstrated that EPS-LM suppresses the expression of the downstream protein NF-κB p65 by inhibiting the upstream pathway components TLR4 and MyD88 and their interactions (Figure 8). By inhibiting the interaction between TLR4 and MyD88, EPS-LM blocks the activation of NF-κB p65. NF-κB p65 is a key subunit of the NF-κB transcription factor complex, responsible for regulating the expression of multiple pro-inflammatory genes [25]. By inhibiting the activation of NF-κB, EPS-LM significantly reduces the production of pro-inflammatory cytokines, thereby alleviating inflammatory responses.

Oxidative stress is a key contributor to cellular damage and inflammation, often exacerbating the effects of inflammatory stimuli like LPS. Excessive ROS can lead to lipid peroxidation, protein oxidation, and DNA damage, which further amplify the inflammatory response and contribute to chronic inflammation and tissue damage [26,27]. By suppressing the TLR4/MyD88/NF-κB signaling pathway, EPS-LM not only reduces the production of pro-inflammatory cytokines but also lowers ROS levels, thereby providing a dual protective effect (Figure 8). The reduction in ROS by EPS-LM is particularly significant because oxidative stress and inflammation are closely linked processes. ROS can activate various signaling pathways, including NF-κB, which, in turn, promotes the expression of pro-inflammatory genes. This creates a vicious cycle where inflammation leads to increased ROS production, and ROS further amplifies the inflammatory response [28]. By breaking this cycle through the inhibition of the TLR4/MyD88/NF-κB pathway, EPS-LM effectively mitigates both oxidative stress and inflammation. Furthermore, the antioxidant properties of EPS-LM help maintain cellular redox balance. This is crucial for the proper functioning of cellular processes and the prevention of apoptosis (programmed cell death) triggered by oxidative damage. By preserving cellular integrity and function, EPS-LM supports the overall health and viability of cells exposed to inflammatory stimuli.

EPS-LM showed a unique and potentially superior ability to suppress both oxidative stress and inflammation compared to other well-known anti-inflammatory agents. For instance, nonsteroidal anti-inflammatory medicines (NSAIDs), such as ibuprofen and aspirin, primarily reduce inflammation by inhibiting cyclooxygenase (COX) enzymes, but their impact on ROS levels is limited [29]. Corticosteroids, like dexamethasone, effectively suppress pro-inflammatory cytokine production by inhibiting the NF-κB pathway, yet they do not directly target ROS, and long-term use can lead to side effects such as immunosuppression and metabolic disturbances [30]. In contrast, antioxidants like N-acetylcysteine (NAC) and vitamin E have been shown more effective in scavenging ROS but less in modulating the key inflammatory pathways like TLR4/MyD88/NF-κB [31,32]. EPS-LM was active not only in reducing ROS levels but also inhibiting the TLR4/MyD88/NF-κB signaling pathway, thus breaking the vicious cycle where oxidative stress and inflammation exacerbate each other [33].

This dual action of anti-inflammation and anti-oxidation through the same signaling pathway highlights the multifaceted protective effects of EPS-LM. The molecular weight and composition of EPS-LM, as identified in this study, provide further insights into its anti-inflammatory potential. The high MW (6.58 × 10^5^ Da) and significant protein content (30 wt%) suggest the presence of complex polysaccharide–protein formations known to enhance biological activity. Compared to previous findings [6], our study identified a higher protein content and a different monosaccharide composition, with a higher prevalence of mannose and the presence of rhamnose and glucosamine. These components interact with specific receptors on immune cells, such as Toll-like receptors. These interactions are critical for regulating immune responses and lowering inflammation. Larger polysaccharide molecules often have more complex and branched structures, enhancing their capacity to engage with immune cell receptors. Furthermore, polysaccharide–protein complexes add to this complexity by possibly providing numerous binding sites for receptor engagement and promoting a stronger anti-inflammatory response [5,34,35]. The FT-IR spectrum analysis further confirms the structural complexity of EPS-LM, highlighting the presence of numerous functional groups associated with bioactivity. The similarities in FT-IR spectra between our study and previous research on EPS-LM from Cs-HK1 underscore the stability and consistency of the fermentation process, while the minor differences observed, such as slight shifts in the FT-IR peaks corresponding to functional groups (e.g., carboxyl, hydroxyl, or glycosidic bonds), might account for variations in bioactivity, particularly in terms of antioxidant and anti-inflammatory effects.

Our findings demonstrate the potent anti-inflammatory and antioxidant properties of EPS-LM, primarily through its modulation of the TLR4/MyD88/NF-κB signaling pathway. This dual action highlights the potential of EPS-LM as a holistic therapy for inflammatory conditions. The effectiveness of similar polysaccharides like ASP-Ag-AP and GLP in reducing colitis via the TLR4/MyD88/NF-κB pathway suggests that EPS-LM, which also exerts anti-inflammatory effects through this pathway, may have comparable protective benefits against inflammatory conditions such as colitis. While our study focused on LPS-stimulated RAW 264.7 cells, the shared mechanism of action indicates that EPS-LM might be beneficial in managing various inflammatory diseases. By modulating the TLR4/MyD88/NF-κB pathway, EPS-LM reduces inflammation and alleviates oxidative damage, demonstrating its potential as a holistic therapy for inflammatory conditions. Future research should be focused on in vivo studies using animal models of inflammation, such as colitis or arthritis, to verify the bioavailability, efficacy, and safety of EPS-LM in complex physiological environments. Following successful animal studies, early-phase clinical trials are essential to assess the safety, tolerability, and efficacy of EPS-LM in humans, particularly in patients with inflammatory disorders such as inflammatory bowel disease (IBD) or rheumatoid arthritis. Moreover, combination therapy studies should investigate whether EPS-LM can enhance the effects of existing anti-inflammatory or antioxidant treatments without increasing side effects.

## 5. Conclusions

The present study has positively detected the specific activity of EPS-LM in inhibiting the TLR4/MyD88/NF-κB signal pathway, which is pivotal for the LPS-induced pro-inflammatory responses in the macrophage cells. EPS-LM has also been shown to suppress the LPS-induced ROS production in the macrophage cell culture. These altogether revealed the multifaceted anti-inflammatory effects of EPS-LM, making it a promising candidate for further research and development in the treatment of various inflammatory and oxidative stress-related conditions, offering a comprehensive approach to managing inflammation and protecting cells from damage. However, while this study highlights the potential of EPS-LM as a therapeutic agent, further in vivo and clinical research is crucial to fully establish its efficacy and safety in humans.

## Figures and Tables

**Table 1:** Chemical composition and molecular weight of Cs-HK1 EPS-LM. Original GPC results shown in Supplemental Data Figure S1.

Sugar (wt%)	Protein (wt%)		Molecular Weight (Da)	
18.71 + 2.27	30.00 + 0.05		6.58 × 10^5^ (±3.32%)	
Monosaccharide composition (molar ratio)				
Mannose	Glucose	Galactose	Rhamnose	Glucosamine
2.191	1.286	1	0.325	0.246

## Data Availability

The original contributions presented in the study are included in the article, further inquiries can be directed to the corresponding author.

## Supplementary Materials

The following are available online at https://www.mdpi.com/article/10.3390/nu16223885/s1, Figure S1: GPC profiles of EPS-LM.

## References

[1] Nathan C., Ding A. (2010). Nonresolving inflammation. Cell.

[2] Medzhitov R. (2010). Inflammation 2010: New adventures of an old flame. Cell.

[3] Chen P.X., Wang S.A., Nie S.P., Marcone M. (2013). Properties of *Cordyceps sinensis*: A review. J. Funct. Foods.

[4] Leung P.H., Wu J.Y. (2007). Effects of ammonium feeding on the production of bioactive metabolites (*cordycepin* and *exopolysaccharides*) in mycelial culture of a Cordyceps sinensis fungus. J. Appl. Microbiol..

[5] Li L.Q., Song A.X., Yin J.Y., Siu K.C., Wong W.T., Wu J.Y. (2020). Anti-inflammation activity of exopolysaccharides produced by a medicinal fungus Cordyceps sinensis Cs-HK1 in cell and animal models. Int. J. Biol. Macromol..

[6] Li L.Q., Song A.X., Wong W.T., Wu J.Y. (2021). Isolation and Assessment of a Highly-Active Anti-Inflammatory Exopolysaccharide from Mycelial Fermentation of a Medicinal Fungus Cs-HK1. Int. J. Mol. Sci..

[7] Guo C., Guo D., Fang L., Sang T., Wu J., Guo C., Wang Y., Wang Y., Chen C., Chen J. (2021). Ganoderma lucidum polysaccharide modulates gut microbiota and immune cell function to inhibit inflammation and tumorigenesis in colon. Carbohydr. Polym..

[8] Zou Y.F., Li C.Y., Fu Y.P., JiZe X.P., Zhao Y.Z., Peng X., Wang J.Y., Yin Z.Q., Li Y.P., Song X. (2023). Angelica sinensis aboveground part polysaccharide and its metabolite 5-MT ameliorate colitis via modulating gut microbiota and TLR4/MyD88/NF-kappaB pathway. Int. J. Biol. Macromol..

[9] Xiao S., Li W.W., Zhang P., Zhang G.W., Lin H.W., Hu X. (2024). Mechanisms of food-derived bioactive compounds inhibiting TLR4 activation and regulating TLR4-mediated inflammation: A comprehensive review and future directions. Food Biosci..

[10] Xu G., Yu Z.P., Zhao W.Z. (2024). Immunomodulation effects of isochlorogenic acid a from apple on RAW264.7 cells *via* modulation of TLR2 and TLR4 target proteins. Food Biosci..

[11] Kawai T., Akira S. (2010). The role of pattern-recognition receptors in innate immunity: Update on Toll-like receptors. Nat. Immunol..

[12] Lu Y.C., Yeh W.C., Ohashi P.S. (2008). LPS/TLR4 signal transduction pathway. Cytokine.

[13] Wei X., Sun W., Zhu P., Ou G., Zhang S., Li Y., Hu J., Qu X., Zhong Y., Yu W. (2022). Refined polysaccharide from Dendrobium devonianum resists H1N1 influenza viral infection in mice by activating immunity through the TLR4/MyD88/NF-kappaB pathway. Front. Immunol..

[14] Leung P.H., Zhao S., Ho K.P., Wu J.Y. (2009). Chemical properties and antioxidant activity of exopolysaccharides from mycelial culture of Cordyceps sinensis fungus Cs-HK1. Food Chem..

[15] Zhao Z.C., Huang L.X., Dong X.L., Wu J.Y. (2024). Evaluation of Three-Phase Partitioning for Efficient and Simultaneous Isolation of Immunomodulatory Polysaccharides and Proteins from *Lentinula edodes* Mushroom. Food Bioprocess Technol..

[16] Li J.H., Gu F.T., Yang Y., Zhao Z.C., Huang L.X., Zhu Y.Y., Chen S., Wu J.Y. (2024). Simulated human digestion and fermentation of a high-molecular weight polysaccharide from Lentinula edodes mushroom and protective effects on intestinal barrier. Carbohydr. Polym..

[17] Barltrop J.A., Owen T.C., Cory A.H., Cory J.G. (1991). 5-(3-Carboxymethoxyphenyl)-2-(4,5-dimethylthiazolyl)-3-(4-sulfophenyl)te trazolium, inner salt (mts) and related analogs of 3-(4,5-dimethylthiazolyl)-2,5-diphenyltetrazolium bromide (mtt) reducing to purple water-soluble formazans as cell-viability indicators. Bioorganic Med. Chem. Lett..

[18] Kleinbongard P., Rassaf T., Dejam A., Kerber S., Kelm M. (2002). Griess method for nitrite measurement of aqueous and protein-containing samples. Methods in Enzymology.

[19] Alia M., Ramos S., Mateos R., Bravo L., Goya L. (2005). Response of the antioxidant defense system to tert-butyl hydroperoxide and hydrogen peroxide in a human hepatoma cell line (HepG2). J. Biochem. Mol. Toxicol..

[20] Burckhardt C.J., Minna J.D., Danuser G. (2021). Co-immunoprecipitation and semi-quantitative immunoblotting for the analysis of protein-protein interactions. Star Protoc..

[21] Akira S., Takeda K. (2004). Toll-like receptor signalling. Nat. Rev. Immunol..

[22] Liu T., Zhang L.Y., Joo D., Sun S.C. (2017). NF-κB signaling in inflammation. Signal Transduct. Target. Ther..

[23] Barnes P.J., Larin M. (1997). Mechanisms of disease-Nuclear factor-kappa b-A pivotal transcription factor in chronic inflammatory diseases. N. Engl. J. Med..

[24] Hayden M.S., Ghosh S. (2011). NF-κB in immunobiology. Cell Res..

[25] Zhang S.Y., Mao C.W., Liu R.W., Zeng X.A., Lin S.Y. (2024). Pulsed electric field (PEF) activates immune activity in RAW 264.7 macrophages by altering pine nut peptide-TLR4 binding sites. Food Biosci..

[26] Zhang Y., Zhang C.C., Luo M.H., Yang S.H., Wang Y.Z., Xu S., Xu Q.R. (2024). Musa basjoo Sieb polysaccharide improves inflammation in RAW264.7 cells and zebrafish colitis. Food Biosci..

[27] Shin H.Y., Kim Y.S., Ha E.J., Koo J.P., Jeong W.B., Joung M.Y., Shin K.S., Yu K.W. (2024). Anti-inflammatory action and associated intracellular signaling of Centella asiatica extract on lipopolysaccharide-stimulated RAW 264.7 macrophage. Food Biosci..

[28] Abbas Z., Tong Y.C., Zhang J., Wang J.Y., Guo H.N., Cheng Q., Marhaba, Zhou Y.C., Ahmad B., Wei X.B. (2024). Enhancing the antioxidant and anti-inflammatory potentials of mulberry-derived postbiotics through submerged fermentation with *B. subtilis* H4 and *B. amyloliquefaciens* LFB112. Food Biosci..

[29] Vane J.R., Botting R.M., Sinzinger H., Samuelsson B., Vane J.R., Paoletti R., Ramwell P., Wong P.Y.K. (1997). Mechanism of action of anti-inflammatory drugs. Recent Advances in Prostaglandin, Thromboxane, and Leukotriene Research.

[30] Barnes P.J. (2006). How corticosteroids control inflammation: Quintiles prize lecture 2005. Br. J. Pharmacol..

[31] Zafarullah M., Li W.Q., Sylvester J., Ahmad M. (2003). Molecular mechanisms of *N-acetylcysteine* actions. Cell. Mol. Life Sci..

[32] Brigelius-Flohé R., Davies K.J.A. (2007). Is vitamin E an antioxidant, a regulator of signal transduction and gene expression, or a ‘junk’ food?. Free. Radic. Biol. Med..

[33] Mittal M., Siddiqui M.R., Tran K., Reddy S.P., Malik A.B. (2014). Reactive Oxygen Species in Inflammation and Tissue Injury. Antioxid. Redox Signal..

[34] Willment J.A., Brown G.D. (2008). C-type lectin receptors in antifungal immunity. Trends Microbiol.

[35] Brown G.D., Gordon S. (2003). Fungal β-glucans and mammalian immunity. Immunity.

