# Supplementary figures and images for: Anti-Inflammatory Effects of Cordyceps Cs-HK1 Fungus Exopolysaccharide on Lipopolysaccharide-Stimulated Macrophages via the TLR4/MyD88/NF-κB Pathway
Source: Nutrients. 2024 Nov 14;16(22):3885. doi: 10.3390/nu16223885 (PMC11597393; doi:10.3390/nu16223885)

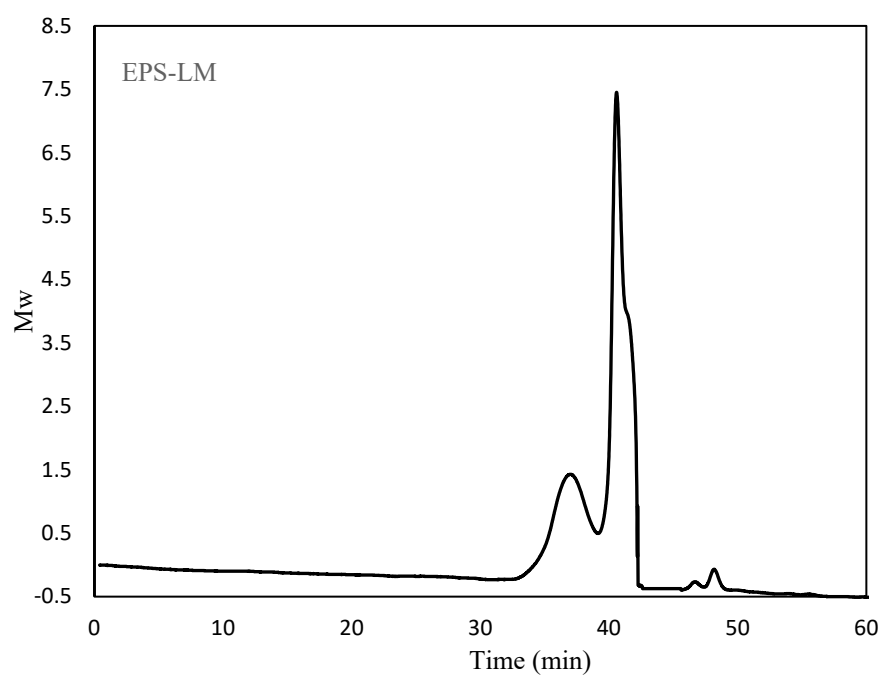

Figure S1. GPC profiles of EPS-LM

Supplement: Supplementary file 1 [file nutrients-16-03885-s001.zip › nutrients-3306101-supplementary.pdf]
